# Correction: Glymphatic inhibition exacerbates tau propagation in an Alzheimer’s disease model

**DOI:** 10.1186/s13195-024-01624-3

**Published:** 2024-11-28

**Authors:** Douglas M. Lopes, Jack A. Wells, Da Ma, Lauren Wallis, Daniel Park, Sophie K. Llewellyn, Zeshan Ahmed, Mark F. Lythgoe, Ian F. Harrison

**Affiliations:** 1 Centre for Advanced Biomedical Imaging, Department of Imaging, Division of Medicine, University College London, Paul O’Gorman Building, 72 Huntley Street, London, WC1E 6DD UK; 2 Department of Internal Medicine, Section of Gerontology and Geriatric Medicine, Wake Forest University School of Medicine, Winston-Salem, NC USA; 3 Neuroscience Next Generation Therapeutics (NGTx), Eli Lilly and Company, Cambridge, MA USA

**Correction** **:** **Alz Res Therapy 16, 71 (2024)**

**https://doi.org/10.1186/s13195-024-01439-2**

Following publication of the original article [1], an error was noticed in the methods section. For the intracerebral infusion of brain extracts into the hippocampus, these were delivered – 2.5 mm anteroposterior to bregma, not + 2.5 mm as the original article indicated.

The original article [1] has been updated.
